# Safety of Intranasal Ketamine for Reducing Uncontrolled Cancer-Related Pain: Protocol of a Phase I/II Clinical Trial

**DOI:** 10.2196/12125

**Published:** 2019-04-30

**Authors:** Jack W Shteamer, R Donald Harvey, Boris Spektor, Kimberly Curseen, Katherine Egan, Zhengjia Chen, Theresa W Gillespie, Roman M Sniecinski, Vinita Singh

**Affiliations:** 1 Department of Anesthesiology Emory University Atlanta, GA United States; 2 Department of Pharmacology Emory University Atlanta, GA United States; 3 Department of Hematology and Medical Oncology Emory University Atlanta, GA United States; 4 Department of Medicine Emory University Atlanta, GA United States; 5 Department of Biostatistics and Bioinformatics Emory University Atlanta, GA United States; 6 Biostatistics and Bioinformatics Shared Resource Winship Cancer Institute Emory University Atlanta, GA United States; 7 Department of Surgery Emory University Atlanta, GA United States

**Keywords:** cancer pain, intranasal ketamine, chronic pain

## Abstract

**Background:**

Approximately 12 million Americans are affected with cancer. Of these, 53% experience pain at all stages of cancer. Pain may remain uncontrolled despite high-dose opioid therapy, and opioids have many well-documented harmful side effects. Intranasal ketamine has been shown to be effective in controlling breakthrough noncancer pain in a double-blind randomized control trial (DBRCT) by Carr et al in 2003 as well as to help with depression in a DBRCT by Lapidus et al in 2014. We seek to obtain preliminary data on the safety, feasibility, and utility of this novel technique for the treatment of uncontrolled cancer pain.

**Objective:**

This study aimed to obtain preliminary data via a clinical trial addressing the safety, feasibility, pharmacokinetics, and pharmacodynamics of intranasal ketamine. These initial findings will be applied to a subsequent trial to determine the effectiveness and associated toxicities of ketamine in a larger sample of cancer patients and to address the compelling need to identify new, successful management therapies for cancer pain.

**Methods:**

This is an institutional review board– and investigational new drug–approved, prospective phase I/II trial to investigate the safety and use of intranasal ketamine in patients with uncontrolled pain related to cancer or cancer treatment. Informed consent will be obtained prior to all study procedures. All patients will be assigned to the same investigational treatment arm. After patient selection via inclusion/exclusion criteria, patients will be seen over 5 visits, with each visit conducted 2-7 days apart. Patients will be administered ketamine on visits 1-4 and monitored for 240 minutes with continuous pulse oximetry and regular blood pressure checks. Blood samples as well as patient-reported outcomes will be collected at set time points at baseline and after drug delivery. Patients will receive 10 mg intranasal ketamine on visit 1, 10 mg intravenous ketamine on visit 2, 30 mg intranasal ketamine on visit 3, and 50 mg intranasal ketamine on visit 4. On visit 5, an addition blood sample will be drawn.

**Results:**

As of March 2019, enrollment is in progress, and a total of 7 subjects have completed the study. Enrollment is expected to be completed by April 2019. Final data analysis will commence soon after, and the results are expected to be submitted for publication in 2019.

**Conclusions:**

If intranasal ketamine can be utilized for pain control in cancer patients, it could provide superior analgesia and better quality of life, without the risk of significant respiratory depression and constipation associated with opioid medications. These findings will be an important initial step toward testing the effectiveness of intranasal ketamine as a nonopioid medication for cancer pain and as potential maintenance outpatient therapy.

**Trial Registration:**

ClinicalTrials.gov NCT03146806; https://clinicaltrials.gov/ct2/show/NCT03146806.

**International Registered Report Identifier (IRRID):**

DERR1-10.2196/12125

## Introduction

### Background and Rationale

About 11.9 million Americans are affected with cancer, of which 53% experience pain at all stages of cancer. This proportion increases to 58%-69% among patients with advanced cancer [1]. Depression often coexists with cancer pain. Patients with cancer pain often require high dosages of opioids that make them too sedated to effectively participate in day-to-day activities and have a good quality of life. Occasionally, even after high-dose opioids, their pain remains uncontrolled in the setting of opioid tolerance or opioid-unresponsive pain. An epidemiological study revealed that 10%-15% of these patients fail to achieve acceptable pain relief with opiates alone or in combination with conventional adjuvant analgesics [2-4].

In the search for improved therapies for chronic cancer pain, medications with novel mechanisms of action have been sought. One such promising pharmacologic approach is ketamine. Ketamine is a Food and Drug Administration (FDA)–approved anesthetic with amnesic, analgesic, dissociative, and sedative properties. It is unique among anesthetic agents, as it does not depress the cardiovascular and respiratory systems. Ketamine is a noncompetitive, antagonist of *N*-methyl-D-aspartate (NMDA) receptors that blocks the NMDA channel in the open state by binding to the phencyclidine site located within the lumen of the channel. Antagonism of NMDA receptors produces antinociception of persistent or neuropathic pain in animal models and analgesia in pain states in humans. The NMDA receptor is believed to play a role in the development of opioid tolerance, and ketamine has been shown to prevent fentanyl-induced hyperalgesia and subsequent acute morphine tolerance in a rat model [5]. Ketamine also interacts at a number of other receptor sites to block pain, including voltage-sensitive calcium channels. Some other effects of ketamine are depression of sodium channels, modulation of cholinergic neurotransmission, and inhibition of serotonin and norepinephrine uptake. Ketamine also interacts with kappa and mu opioid receptors; however, in humans, naloxone, an opioid antagonist, does not antagonize the analgesic effects of ketamine. Safety and efficacy of ketamine as an anesthetic and analgesic agent are well documented [6-8]. Ketamine is not labeled as an analgesic agent by the FDA. Low (subanesthestic) doses of ketamine have minimal adverse impact upon cardiovascular or respiratory function but produce analgesia and modulate central sensitization, hyperalgesia, and opioid tolerance. Ketamine is typically administered intravenously, but the intravenous route is not convenient for use in an ambulatory setting. Although ketamine has been administered orally, its oral bioavailability is low due to hepatic first pass, limiting the application of this route for chronic use. The intranasal route has several advantages such as higher bioavailability due to avoidance of first-pass hepatic metabolism, no need for venous access, ability to repeat doses quickly, and rapid absorption [9-10].

Intranasal ketamine is effective in controlling breakthrough pain, as shown in a double-blind randomized controlled trial (DBRCT) by Carr et al in 2003 [11], as well as in helping with depression, as shown in a DBRCT by Lapidus in 2014 [12]. The Carr study [11] was a DBRCT including 20 patients with breakthrough pain and noted pain relief within 10 min, lasting up to 60 minutes with 10-50 mg of intranasal ketamine (2.65 point average decrease on the Numerical Pain Rating Score [NPRS] scale). The pain scores were only recorded until 60 minutes after drug administration, and the patients were allowed to determine the dose themselves, without precise description for the dose in the study. The study by Lapidus et al [12] was performed in 20 patients with major depression and noted significant improvement in depressive symptoms (7.6 point average decrease on the Montgomery-Asberg Depression Rating Scale [MADRS] scale) in 44% of patients at 24 hours after administration of 50 mg intranasal ketamine. There are limited data regarding the use of ketamine as an adjuvant to opioids for the management of *cancer* pain [13]. We seek to obtain preliminary data on the safety, feasibility, and utility of this novel technique for the treatment of uncontrolled cancer pain.

### Objectives

If intranasal ketamine can be utilized for pain control in patients with cancer, it could provide superior analgesia and a better quality of life without the risk of significant respiratory depression associated with opioid medications. We seek to obtain preliminary data via a clinical trial addressing the safety, feasibility, and utility of this novel technique for the treatment of persistent uncontrolled cancer pain. These findings would be an important initial step toward testing the effectiveness of intranasal ketamine as a nonopioid medication for cancer pain and as potential maintenance outpatient therapy. These initial findings will be applied to a subsequent trial to determine the effectiveness and associated toxicities of ketamine in a larger sample of cancer patients and to address the compelling need to identify new, successful management therapies for cancer pain.

## Methods

### Trial Design

**Figure 1:**
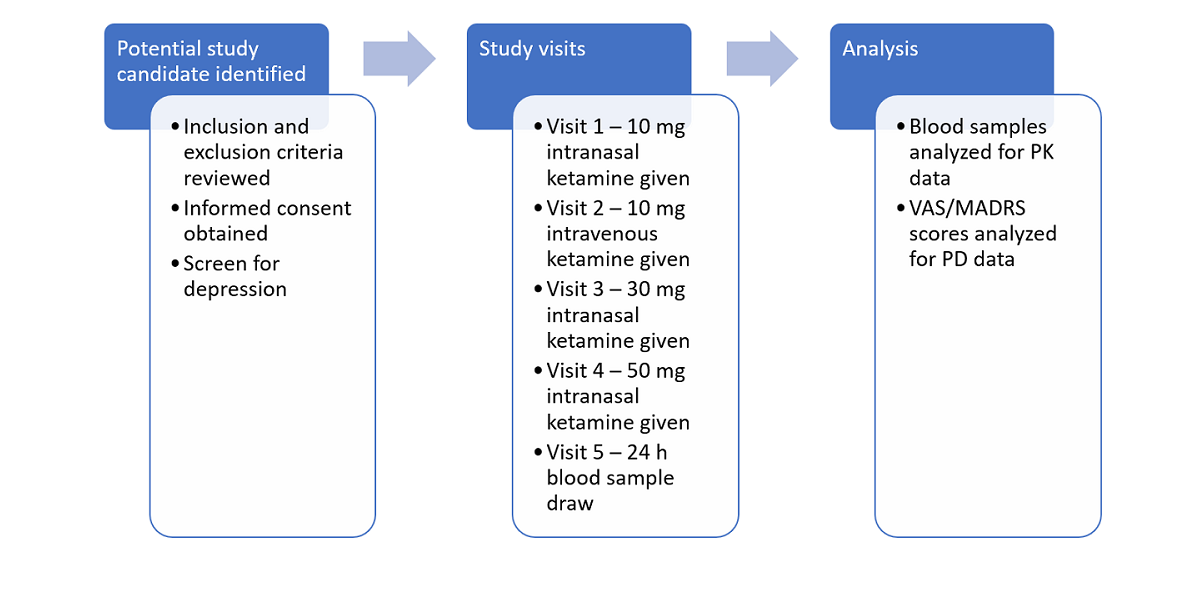
Participant timeline. PK: pharmacokinetic; PD: pharmacodynamic; VAS: Visual Analoge Scale; MADRS: Montgomery Asberg Depression Rating Scale.

This is a prospective clinical trial aimed to investigate the use of intranasal ketamine in patients with uncontrolled pain related to cancer or cancer treatment (Figure 1). All patients will be assigned to the same investigational treatment arm.

### Participants, Interventions, and Outcomes

#### Study Setting

The trial will be conducted at the Phase I Unit of the Winship Cancer Institute, an academic hospital in Atlanta, Georgia, United States. Subjects will be recruited at the supportive oncology, oncology, and pain clinics at an academic institution located in Atlanta, Georgia. Subjects may be identified and contacted via telephone with information about the study prior to their next clinic appointment in order to allow time for them to consider the study.

#### Eligibility Criteria

Patients will be eligible to participate if they are (1) men or women of at least 18 years of age; (2) patients with uncontrolled pain related to cancer or cancer treatment (uncontrolled pain will be defined as (i) pain that persists for more than 7 days and is rated ≥4 on the NPRS or (ii) use of breakthrough medication more than 4 times in 24 hours or receiving treatment with oral morphine ≥50 mg/day); (3) patients who are able to follow-up in person during the trial; (4) patients on a stable analgesic regimen for >7 days without escalation during the study period, receiving rescue or immediate-release medication every 3 or more hours; (5) patients who are willing and able to maintain a daily pain diary; (6) patients who are able to understand written and verbal English; and (7) patients who weigh ≥50 kg.

Patients will be excluded from the study if they have any of the following:

- Transportation issues interfering with return study visits
- High disposition of laryngospasm or apnea
- Severe cardiac disease
- Conditions where significant elevations in blood pressure would be a serious hazard
- Stage 2 or higher hypertension (systolic blood pressure>160 mmHg or diastolic blood pressure>100 mmHg)
- Baseline tachycardia (heart rate>100 beats/minute)
- History of seizures, elevated intracranial pressure, or cerebrospinal fluid obstructive states (eg, severe head injury and central congenital or mass lesions)
- Conditions that may increase intraocular pressure (eg, glaucoma and acute globe injury)
- History of uncontrolled depression or other psychiatric comorbidity with psychosis
- History of liver disease
- History of interstitial cystitis
- History of nasal or sinus anomalies or dysfunction (eg, allergic or infectious rhinitis)
- Lesions of the nasal mucosa
- Pregnancy, ongoing nursing, and childbearing potential but no use of contraception known to be highly effective. Highly effective contraception methods include a combination of any two of the following: use of oral, injected, or implanted hormonal methods of contraception or placement of an intrauterine device or intrauterine system; barrier methods of contraception such as condom or occlusive cap (diaphragm or cervical/vault caps) with spermicidal foam/gel/film/cream/vaginal suppository; total abstinence; and male/female sterilization
- Illicit substance abuse within the past 6 months
- Documented history of medication abuse/misuse (eg, unsanctioned dose escalation and broken opioid agreement)
- Clinical requirement for medications that are concurrent inducers or strong inhibitors of CYP3A4. CYP3A4 substrates are allowed.
- Porphyria (the possibility of triggering a porphyric reaction)
- Severe active anemia (hemoglobin level<8 g/dL documented by laboratories; blood drawn within 3 months of the first study treatment)
- History of difficult intravenous access
- Intractable vomiting

#### Interventions

Written informed consent will be obtained prior to the conduct of study procedures, either while recruiting subjects prior to study visit 1 or during the first study visit prior to the beginning of the study. Subjects must meet all study eligibility criteria on visit 1 prior to study treatment. Intranasal ketamine will be prepared from a 100 mg/mL vial to be delivered nasally with a nasal atomizer. We will deliver 0.1 mL to provide 10 mg of atomized ketamine. Alternating nares will be used to deliver 0.1 mL at a time until the full dose is delivered. Christensen et al [14] reported the safety of intranasal doses of 10 mg, 30 mg, and 50 mg ketamine. Therefore, for the purpose of this study, 10 mg, 30 mg, and 50 mg of intranasal ketamine will be administered. Each visit will be conducted 2-7 days apart to avoid any accumulation of study doses. Patients will be asked to return to the Phase I Unit for a total of five study visits. All study visits will take place within 6 weeks or less.

On the first study visit, 10 mg of intranasal ketamine will be given to make sure that the study patients are able to tolerate a small dose of intranasal ketamine. On the second visit, 10 mg of intravenous ketamine will be given to help establish bioavailability of intranasal ketamine, with patients serving as their own controls. On the third and fourth visits, higher doses of ketamine, specifically, 30 mg and 50 mg, respectively, will be given, if the patients did not have severe adverse events with the smaller dosage. On the fifth study visit, no ketamine will be administered.

Licensed study personnel will administer the ketamine. The intranasal ketamine will be administered in the following process: First, the mucosal atomization device (MAD) will be primed, and the appropriate dose will be loaded into the syringe. The patient will be seated in a chair and clear his/her sinuses using a tissue, if needed. The patient’s head will be tilted backward. The MAD will be inserted into a nostril and aimed directly posterior, leveling with the floor of the nare and slightly lateral. A total of 0.1 mL (10 mg) ketamine will be administered. The patient will be asked to keep his/her head tilted backward for 5 minutes, if tolerable, in order to ensure that the medication does not run out of the nares. If medication visibly runs out of the nares or if the patient reports feeling the medication trickling down his/her throat, the event will be noted. If more than 0.1 mL (10 mg) needs to be delivered, steps 3-5 will be repeated in the contralateral nare, alternating until the full dose is delivered. After a short break of 30 seconds, ketamine will be re-administered into the same nare.

Patients may continue to take their usual pain medication as needed. Since the effect of most of the oral immediate-release opioid medications peaks between 30 and 90 minutes, ketamine will be given at least 120 minutes after the last dose of immediate-release opioid medication, so that the reduction in pain score is not confounded with the results of immediate-release opioid medications.

A family member/friend must transport the patient home following drug administration, as driving for 24 hours after drug administration is discouraged. A telephone call will be placed 14 days after the last dose of medication is administered to follow up any ongoing adverse events that occurred during the study period. If there are no ongoing adverse events at the end of visit 5, the subject’s participation will end at that time. Provided all eligibility requirements are met, subjects may be reconsented to participate if they withdrew early, at the discretion of the investigator.

As the study subjects will have uncontrolled pain, it is likely that they might be on opioid medications during the study. Patients may continue their opioid and pain medication as prescribed. The Georgia prescription-monitoring database will be searched by the investigators, and an opioid pill count will be performed at each visit to monitor the opioid intake during the study.

If there is any hospitalization related to the study drug administration, the study will be put on hold for a formal review by the principal investigator and the Data and Safety Monitoring Committee (DSMC). The principal investigator or delegated person will communicate the event details to the DSMC for their review and determination of approval to continue the study. The study will be discontinued if there is more than one hospitalization related to administration of the study drug.

#### Outcomes

##### Primary Outcomes

The primary outcomes are as follows: (1) To conduct a clinical trial of intranasal ketamine in a sample of patients with cancer-related pain, the pharmacokinetics of intranasal ketamine will be measured through analysis of ketamine and its metabolite norketamine to determine pharmacokinetic variables including peak concentration after each dose and route (C_max_), time to peak concentration, total area under the concentration time curve (AUC_0-t_), half-life, and clearance. (2) To evaluate (pharmacodynamic) the effects of intranasal ketamine on patient-reported outcomes such as pain scores, side effects, depression, health-related quality of life, and functional status, the patient-related outcome will be assessed as measured by the NPRS, Side Effect Rating Scale for Dissociative Anesthetics (SERSDA), MADRS, Edmonton Symptom Assessment (ESAS), Eastern Cooperative Oncology Group (ECOG), and Patient-Reported Outcomes Measurement Information System (PROMIS) scales.

##### Secondary Outcomes

To determine the opioid-sparing effect of intranasal ketamine, we will (1) document the use of rescue medications prior to and during the study and (2) evaluate the total opioid consumption prior to and during the study.

##### Measurements

The following assessments will be documented in the patient research study record (Table 1):

- Height and weight at the first study visit
- Pain intensity on the NPRS prior to and at 5, 10, 15, 30, 45, 60, 120, 180, and 240 minutes after medication is given
- Vital signs including heart rate, blood pressure, respiratory rate, and pulse oximetry at baseline and 5, 10, 15, 30, 45, 60, 120, 180, and 240 minutes. Vital signs and pulse oximetry will be monitored continuously for a minimum of 30 minutes after drug administration. Vital signs will be measured more frequently if they are unstable
- The ECOG and ESAS scores at baseline and on each visit
- Sedation level on the Richmond Agitation-Sedation Scale (RASS) scale prior to and at 30, 60, and 240 minutes after medication is given. If the RASS score is between +4 and –3, sedation/delirium assessment on the Confusion Assessment Method for the Intensive Care Unit scale will be performed prior to and at 30, 60, and 240 minutes after medication is given
- Side effects using the SERSDA prior to and at 30, 60, and 240 minutes after medication is given
- Olfactory assessment before and after study drug administration to determine if the study medication has impacted the sense of smell
- Intake of any rescue medication; if taken, we will determine if it was taken at the usual interval or after a longer interval
- Patient response to the intervention if anything besides change in NPRS is reported
- Patient and family education regarding maintaining a pain diary (Multimedia Appendix 11) and reviewing discharge instructions, including when to call the study coordinator or report to the emergency department
- PROMIS functional scale on the first and last days of the study
- Any change in health status during the duration of study
- Opioid medication pill count at the beginning of each visit
- Only for visits 1, 3, and 4: If the patient reports symptoms of a significant upper respiratory tract infection (eg, rhinorrhea or congestion), the study visit will be canceled and rescheduled at a later date
- If changes in the sense of smell are noted, a study investigator will be notified, and additional olfactory assessments will be performed, with the frequency and number of assessments at the discretion of the investigator
- The frequency of the assessments may be increased at the discretion of the investigator

The study nurse will notify the attending physician if any of the following occur:

- Baseline heart rate>100 beats/minute, blood pressure>160/100 mmHg, or pain score≤5 on the NPRS. The physician will make the decision of whether to proceed with the study visit or cancel it based on the inclusion/exclusion criterion
- Any side effects or unexpected results or the vital signs change more than 20% from baseline (heart rate<50 beats/minute or >110 beats/minute, systolic blood pressure<90 mmHg or >180 mmHg, diastolic blood pressure<30 mmHg or >100 mmHg, respiratory rate<8 or >24 breaths/minute, or oxygen saturation <90%) after the study medication is given
- Sedation on the RASS scale is between –1 and –5 at any time
- Positive results for delirium on the Confusion Assessment Method for the Intensive Care Unit scale at any time
- Presence of any possible side effects from the study drug administration

Patients will be deemed eligible for discharge after the 240-minute assessment if their vital signs are within 10% of the baseline values; their RASS score is between +1 and –1; and side effects, as noted on the SERSDA scale, are ≤2 or no more than 1 point greater than their baseline value (the investigator must be notified if the value is greater than 2).

If the patient does not meet these criteria at the end of the 240-minute assessments, the study nurse will contact the research physician for further evaluation and assessment.

### Sample Size

A sample size of 7 from a population of 20 (in the study by Carr et al [11]) yields a power of 91% to detect an NPRS difference of –2.7 between the null hypothesis mean of 0.0 and the alternative hypothesis mean of 2.7, with an estimated SD of 1.9 and a significance level (alpha) of .05 using a two-sided Wilcoxon test, assuming that the actual distribution is normal.

A sample size of 7 patients is needed based on statistical analysis observed in the study by Carr et al [11]. We will include 10 patients to account for the possibility that the observed pain reduction in this study may be different from that reported by Carr et al [11], as in this study, patients were given ketamine for breakthrough pain, not for baseline pain. We will enroll up to 25 patients in the study to account for potential dropouts. If a higher dropout rate is observed, we will enroll more patients until we have at least 10 patients who complete the entire study.

Any subject who does not complete the intravenous dosing and at least one intranasal dose will be replaced and followed up for safety, as needed, or receive a telephone call at 14 days. Enrolled subjects who discontinue the study early will be replaced. If a subject misses more than one treatment visit during the study period, the subject will be withdrawn and replaced to achieve a minimum of 10 subjects who are maximally treated with study medication (four study visits).

**Table 1:** Assessment schedule.

Assessments	Screening	Visit 1	Visit 2	Visit 3	Visit 4	Visit 5	Telephone call
Review of inclusion/exclusion	✓						
Informed consent	✓						
Medical history/demographics	✓						
Hemoglobin level (if none in patient records within 3 months of visit 1)		✓					
Urine pregnancy test - within 1 week of planned visit (women of child-bearing potential)		✓	✓	✓	✓	✓	
Depression screening using PHQ-9^a^	✓	✓^b^					
Completion of the PROMIS^c^ questionnaire		✓				✓	
ECOG^d^ scale		✓	✓	✓	✓	✓	
ESAS^e^ scale		✓	✓	✓	✓	✓	
MADRS^f, g, h^		✓	✓	✓	✓	✓	
Height		✓				✓	
Weight		✓	✓	✓	✓	✓	
Intravenous access		✓	✓^i^	✓	✓		
Study medication administration		✓	✓	✓	✓		
Vital Signs (heart rate^j^, blood pressure, respiratory rate, pulse oximetry^k^)		✓^l^	✓^l^	✓^l^	✓^l^	✓^m^	
Blood samples (pharmacokinetics		✓^m^	✓^n, o^	✓^n, o^	✓^n, o^	✓^o^	
Hepatic function test		✓				✓	
Urinalysis		✓				✓	
Pain scores using NPRS^p^		✓^l^	✓^l^	✓^l^	✓^l^	✓^m^	
RASS^q^ scale		✓^r^	✓^r^	✓^r^	✓^r^	✓^m^	
CAM ICU^s^ scale for RASS score between +4 to –3		✓	✓	✓	✓	✓	
SERSDA^t^ scale		✓^r^	✓^r^	✓^r^	✓^r^	✓^m^	
Olfactory assessment - before and after drug administration		✓	✓	✓	✓		
Adverse events		✓	✓	✓	✓	✓	✓^u^
Details of rescue medication use		✓	✓	✓	✓	✓	
Pain diary^v^		✓	✓	✓	✓	✓	
Opioid pill count		✓	✓	✓	✓	✓	

^a^PHQ-9: Patient Health Questionnaire - 9 items.

^b^If not previously recorded during the screening visit or within 3 months of planned visit one, and no history of depression.

^c^PROMIS: Patient-Reported Outcomes Measurement Information System.

^d^ECOG: Eastern Cooperative Oncology Group.

^e^ESAS: Edmonton Symptom Assessment.

^f^MADRS: Montgomery-Asberg Depression Rating Scale.

^g^If screened positive for depression, questionnaire to be administered before medication is given.

^h^Questionnaire to be repeated between 180 and 240 minutes after medication is given.

^i^Two intravenous access points are needed at visit 2—One for study medication and one for blood draws.

^j^Monitoring will occur continuously for a minimum of 30 minutes after drug administration.

^k^Pulse oximetry monitoring will occur continuously for a minimum of 30 minutes after drug administration.

^l^To be recorded at baseline and 5, 10, 15, 30 (±5), 45 (±5), 60 (±5), 120 (±15), and 240 (±15) minutes after medication administration.

^m^On arrival for study visit.

^n^Samples will be obtained at 2, 30 (±5), 60 (±5), and 240 (±15) minutes after medication administration on visits 1-4.

^o^Baseline samples will be drawn on visits 2-5.

^p^NPRS: Numerical Pain Rating Score.

^q^RASS: Richmond Agitation-Sedation Scale.

^r^Assess at baseline and 30 (±5), 60 (±5), and 240 (±15) minutes after medication administration.

^s^CAM ICU: Confusion Assessment Method for the Intensive Care Unit.

^t^SERSDA: Side Effect Rating Scale for Dissociative Anesthetics.

^u^Ongoing adverse events will be followed up by a telephone call 14 days after the last day of study medication administration (±1 day).

^v^Patient to record data throughout study enrollment. Data are collected at final visit.

### Recruitment

Subjects will be recruited at the supportive oncology clinic, oncology clinics, the pain clinic, and the Acute Pain Service at Emory by research coordinators and investigators. Subjects may be identified and contacted via telephone with information about the study prior to their next clinic appointment in order to allow time for them to consider the study.

### Data Collection, Management, and Analysis

#### Data Collection Methods

##### Pharmacokinetics

Blood samples will be drawn at 2, 30, 60, and 240 minutes. Baseline blood samples will be drawn on Visits 2 through 5. The specimen requirements for laboratory testing are 3 mL serum or plasma in EDTA-containing vacuum tubes. We will draw 6 mL blood per sample to allow for adequate serum/plasma samples. The total blood drawn during one visit would be 24-30 mL. Samples will be promptly centrifuged, and serum or plasma will be separated into a plastic cryovial and frozen. The plastic containers will be preservative free. All samples will be sent to National Medical Services within 3 months. The samples are valid for 7 months at –20°C and for 30 days when refrigerated or stored at room temperature. Samples will be destroyed immediately after analysis. Gas chromatography with mass spectrometry analysis of the samples will be performed to determine the concentrations of ketamine and its metabolite, norketamine. Plasma concentration-time curves for ketamine and norketamine will be generated for each subject and compared among subjects for the variables of route and dose, with intravenous administration serving as the basis for comparison.

##### Pharmacodynamics

All assessment forms are provided in Multimedia Appendices 1-10.

Pain will be measured using the NPRS. This scale is the most responsive tool to document pain intensity as compared to the Visual Analogue Scale and Visual Rating Scale for measuring pain and shows higher compliance rates, better responsiveness, easier use, and better applicability than the latter scales [15,16]. In general, improvements of ≤1.5 points for pain severity on NPRS could be seen as clinically irrelevant [17-20]. Above that value, the cutoff point for “clinical relevance” depends on patients’ baseline pain severity and ranges from 2.4 to 5.3 [19-21]. Higher baseline scores require larger raw changes to represent clinically important differences [22].

Patients will maintain a pain diary for the entire study period until the final study visit and record how often they took their usual breakthrough medication during the trial. Patients will log pain scores three times a day.

The 10-item PROMIS questionnaire will be filled out at the first (baseline) and fifth (final) study visit to assess global health.

Participants will fill out a Patient Health Questionnaire - 9 items (PHQ-9) scale during the screening visit or the first study visit. The PHQ-9 scale ranges from 0 to 27 points and can be used for screening of depression. Patients who are on antidepressants or score more than 4 points on the PHQ-9 scale will be assessed for depression during the study. Depression will be assessed based on the MADRS on each visit. This is a 10-item questionnaire designed to be particularly sensitive to treatment effects [23]. Higher MADRS scores indicate more severe depression, and each item yields a score of 0-6 points. The overall score ranges from 0 to 60. Participants who screen positive for depression and are not on any treatment for depression will be offered a psychiatry consult as a part of their routine healthcare, if interested.

The performance status will be assessed by ECOG grading at baseline and throughout the study, which ranges from 0 to 5, where 0 is fully active and 5 is dead.

ESAS will be used to assess 9 common symptoms (pain, tiredness, nausea, depression, anxiety, drowsiness, appetite, well-being, and shortness of breath) experienced by cancer patients. Each symptom is rated from 0 to 10 points on a numerical scale, with 0 indicating that the symptom is absent and 10 indicating that the symptom is at its worst possible severity.

Side effects will be assessed using the SERSDA questionnaire prior to and at 30, 60, and 240 minutes after medication is given. The SERSDA questionnaire rates fatigue, dizziness, nausea, headache, feeling of unreality, changes in hearing, changes in vision, mood change, generalized discomfort, and hallucination on a scale of 0-4 points.

#### Data Management

The privacy of the research subjects will be ensured through the standard procedures for securing research data, including data encryption, limiting data access to trained personnel, and de-identification of subjects. All records identifying the subject will be kept confidential and, to the extent permitted by the applicable laws and regulations, will not be made publicly available. If the results of the study are published, the subject’s identity will remain confidential. Data will be stored in two locations: Hard copies will be kept in a secure and locked location, and soft copies will be stored electronically via a secure server.

#### Statistical Methods

##### Pharmacokinetics

To determine the pharmacokinetic characteristics such as bioavailability, peak effect, and elimination of intranasal ketamine, concentrations of ketamine and its metabolite norketamine will be analyzed.

##### Pharmacodynamics

To determine the pharmacodynamics of intranasal ketamine in terms of patient-related outcomes, pain scores on NPRS will be analyzed using the Wilcoxon signed-rank test, as the study sample size is too small to be analyzed by paired *t* test. A generalized Linear Model will be used to model any time trend for pain score change and to test if the trend is significant. A Wilcoxon signed-rank test will be used to compare any changes in depression severity on MADRS. A Chi-square test will be used to test the presence/absence of any side effects. A Wilcoxon signed-rank test will be used to assess the severity of all side effects (including SERSDA), if needed; to determine any changes in functional status on the PROMIS scale; and to compare any changes in the ESAS and ECOG scores.

##### Pharmacokinetic-Pharmacodynamic Relationships and Opioid-Sparing Effect

To determine the pharmacokinetic-pharmacodynamic relationships of intranasal ketamine delivered, we will compare the timing, degree, and duration of change in pain scores with individual and group pharmacokinetic parameters in order to assess potential relationships between the measures of exposure (C_max_, and AUC_0-t_) and pain relief. Multivariate modeling including patient-specific data (age, sex, weight, body mass index, and morphine equivalents) will be performed using initial pharmacokinetic parameters and goodness-of-fit analyses for compartmental model selection. If applicable, data from vital sign measurement changes will also be used for comparison with pharmacokinetic data.

To determine the opioid-sparing effect of intranasal ketamine, a Chi-square test will be used to compare the frequency of rescue medication use and a Wilcoxon signed-rank test will be used to compare the total opioid consumption.

### Monitoring

#### Data Monitoring

Data will be monitored by the Winship Cancer Institute DSMC. The DSMC oversees internal monitoring functions by reviewing study conduct for consistency with Good Clinical Practice, compliance with federal regulations, and production of high-quality scientific data. The DSMC comprises physician investigators, internal monitors, and administrative staff. Initial study monitoring will occur within 6 months from the date of the first subject accrued, with two of the first five subjects already reviewed. Therefore, subsequent monitoring will occur in 6-month intervals, if any subjects are accrued. The population continuing to receive the intervention will be monitored as determined by the DSMC. At minimum, 10% of the subjects accrued since previous monitoring will be reviewed. An additional subject (or subjects) may be selected based on previously noted monitoring deficiencies or at the DSMC’s discretion. Continued monitoring will occur in 6-month intervals for the population continuing to receive the intervention as determined by the DSMC. The DSMC will review pertinent aspects of the study conduct including patient safety, protocol compliance, data collection, and efficacy. The principal investigator or designee will be responsible for notifying the DSMC of patient accrual and status updates within 2 months of the planned review. The DSMC is independent from the study sponsor; however, the chair of the DSMC (DV) is a coinvestigator. DV steps out of DSMC meetings whenever the study is being discussed, and the vice-chair of the DSMC assumes the role of chair.

#### Harms

The following methods will be used to avoid harms:

1. A monthly meeting of investigators, clinical research coordinators, and regulatory personnel will be held to review the clinical data.
2. Adverse event reporting will utilize the Common Terminology Criteria for Adverse Events, version 5.0. The grading scale for these adverse events is as follows:
  - Grade 1 - Mild: asymptomatic or mild symptoms, clinical or diagnostic observations only, intervention not indicated
  - Grade 2 - Moderate: minimal, local, or noninvasive intervention indicated, limiting age-appropriate instrumental activities of daily living (ADL)
  - Grade 3 - Severe or medically significant but not immediately life-threatening: hospitalization or prolongation of hospitalization indicated, disabling, limiting self-care ADL
  - Grade 4 - Life-threatening consequences: urgent intervention indicated
  - Grade 5 - Death related to an adverse event
  - A serious adverse event is an adverse event or suspected adverse drug reaction that fulfills one of the following criteria: it results in death; it is immediately life-threatening (life-threatening in the definition of serious adverse event refers to an event in which the subject was at immediate risk of death at the time of the event; it does not refer to an event that hypothetically might have caused death if it were more severe); it requires in-subject hospitalization or prolongation of existing hospitalization; it results in persistent or significant disability or incapacity; it is a congenital anomaly or birth defect; it is an important medical event (important medical event in the definition of “serious” refers to an event that may not be immediately life threatening, or result in death or hospitalization, but from medical and scientific judgment, it may jeopardize the subject or require medical or surgical intervention to prevent one of the other outcomes listed above. Examples of such events are intensive treatment in an emergency room or at home for allergic bronchospasm, blood dyscrasias, or convulsions that do not result in hospitalization. Development of cancer or drug dependency/abuse will normally be considered serious as per this criterion)
3. Study progress in terms of enrollment and activity of current patients will be reviewed during the monthly meeting of the investigators, clinical research coordinators, and regulatory personnel. The principal investigator may increase the frequency of this meeting, if necessary.
4. As the investigational new drug sponsor-investigator, unexpected fatal or life-threatening suspected adverse reactions will be reported using the Medwatch FDA form 3500A via FedEx to the FDA no later than 7 calendar days after the initial receipt of information (21CFR 312.32(C)(2)). Additionally, serious unexpected suspected adverse reactions and a clinically important increase in the rate of a serious suspected adverse reaction will be reported no later than 15 calendar days after determining that the information qualifies for reporting (21 CFR 312.32(C)(1)) in paper format using FDA form 3500A. Finally, an annual progress report will be provided within 60 days of the anniversary date that the investigational new drug became active (ie, January 20, 2017 (21 CFR 312.33)).
5. Adverse events will be recorded. An adverse event is defined as any untoward medical occurrence in a patient or clinical investigation subject administered a pharmaceutical product or study treatment and that does not necessarily have a causal relationship with this administration. An adverse event can therefore be any unfavorable and unintended sign (including any abnormal laboratory findings), symptom, or disease temporally associated with the use of a medicinal (investigational) product, irrespective of whether it is related to the medicinal (investigational) product. Adverse events will be captured from the time of the first study drug administration through the fifth study visit. Ongoing adverse events at the fifth visit will be followed with a telephone call 14 days (plus or minus 1 day) after the last medication administration. Adverse events will be reported to the principal investigator by Emory email or telephone. All adverse events will be documented in an adverse event log. The reporting policy of the Emory Institutional Review Board will be followed.
6. The study will be put on hold after any hospitalization that is related to study drug administration for a formal review by the principal investigator and the DSMC. The principal investigator or delegated person will communicate the event details to the DSMC for their review and determination of approval to continue the study. The study will be end if there is more than one hospitalization related to administration of the study drug.
7. The Emory University School of Medicine Department of Anesthesiology will conduct this study according to national rules, regulations, and guidelines governing human clinical research. In addition, procedures cited by the US Code of Federal Regulations (Title 21) will be followed, as these apply to the principles of Good Clinical Practice and approval by the Emory University Institutional Review Board.

#### Auditing

This study will be followed by the Winship Cancer Institute Data Safety Monitoring Committee (DSMC) for local review and confirmation of proper study execution and safety measures. See the “Data Monitoring” section for further details.

### Ethics and Dissemination

#### Research Ethics Approval

Institutional review board approval has been obtained, and the specified policies and procedures will be followed.

#### Protocol Amendments

All protocol changes will first be approved by the DSMC and Institutional Review Board. Approved important protocol changes (eg, changes to eligibility criteria, outcomes, and analyses) will be disseminated to relevant parties (eg, investigators, study staff, trial participants, trial registries, and journals). The following protocol amendments have been made and approved by the institutional review board (all changes reflected in this protocol):

- Amendment 1: Feb 13, 2017 to May 2, 2017 
  - Corrections to Table 1. The assessment schedule has been modified as follows: A negative pregnancy test is required for child-bearing women within 1 week of study drug administration and may therefore require repetition at any point during the study visit period depending on the schedule of the visits. Continuous monitoring will be performed, and the requirement for telemetry will be removed. The word “Opioid” has been added to pill count. ECOG and ESAS have been removed from screening assessments. The missing 10-minute pain score assessments have been added
  - A goals/outcomes section has been expanded
  - More detail has been added to the Statistical Methods section
- Amendment 2: May 2, 2017 to Feb 23, 2018
  - Additional study identification has been included
  - Minor grammatical errors have been corrected
  - Potential subjects may be contacted by telephone in advance of the next scheduled clinic appointment
  - Clarification has been added that eligibility requirements must be confirmed at visit 1. A hemoglobin test may be ordered if required to meet the eligibility criteria in visit 1
  - Replacement/reconsent of subjects has been addressed
  - Requirement to notify the investigator of side effects has been added
  - Treatment strategy for central nervous system side effects has been added
  - Enrollment has been increased up to 25 people in order to achieve 10 treated patients
  - Clarification of the FDA reporting process has been provided
- Amendment 3: Feb 23, 2018 to May 24, 2018
  - Visit days may be up to 7 days apart versus the previous 5 days apart, and the total time to study completion may be up to 6 weeks
  - The follow-up phone call will only occur if there are ongoing adverse events on visit 5
  - Clarification of the telephone call visit has been added
  - Use of the CAM ICU scale has been corrected
  - Subject monitoring parameters for patients #15, #16, and #17 have been added
  - A more detailed description of study drug-administration process has been added
  - Requirement of research physician notification for baseline pain score value has been added
  - Morphine equivalent requirement has been reduced from 100 to 50 in the eligibility criteria
- Amendment 4: May 24, 2018 to Jan 2, 2019
  - The acceptable pain score for study inclusion has been reduced from 6 to 4 points

#### Consent or Assent

Written informed consent will be obtained by a research team member delegated to do so by the principal investigator, prior to conducting any research procedures. This process will include a discussion of risks and benefits as well as answering patient questions. If patients are able to provide their own medical history, they will be deemed capable of providing informed consent for research participation. The process will be free of coercion. Study patients will be paid a small amount as reimbursement for travel expenses for each visit completed. They can withdraw from the study at any time without penalty to their ongoing care.

#### Confidentiality

The privacy of the research subjects will be ensured through the standard procedures for securing research data. All records identifying the subject will be kept confidential and, to the extent permitted by the applicable laws and regulations, will not be made publicly available. Subjects will be identified by a study number. If the results of the study are published, the subject’s identity will remain confidential.

#### Access to Data

Access to study data will be limited to trained study personnel specifically delegated to do so. Logs of personnel training and role delegations will be maintained.

#### Ancillary and Posttrial Care

A telephone call will be placed 14 days after the last dose of medication is administered, to follow up on any ongoing adverse events that occurred during the study period. If there are no ongoing adverse events at the end of visit 5, the subject’s participation will end at that time.

If a patient becomes ill or injured due to participation in the study, Emory University will help the patient obtain medical treatment. Emory University has not, however, set aside any money to pay for this medical treatment. The only exception is if it is proven that the patient’s injury or illness is directly caused by the negligence of an Emory employee. “Negligence” is the failure to follow the standard duty of care.

If a patient becomes ill or injured due to participation in this study, the patient’s insurer will be billed for the associated treatment costs. If the patient does not have insurance, or if the insurer does not pay, the patient will have to pay the costs.

A compensation of US $50 per visit will be provided to each patient for each completed visit as reimbursement for travel expenses to and from the institution.

#### Dissemination Policy

Results of the trial will be submitted for publication to major peer-reviewed scientific and medical journals. If no peer-reviewed journal accepts the manuscript for publication, it will be submitted to an open access journal for publication. The results will also be reported in the Clinicaltrials.gov database. Publication will be listed in PUBMED Central for public access. The protocol will be submitted for publication to peer-reviewed journals.

## Results

Initial funding was approved by the Emory University Department of Anesthesiology in August 2016. Investigational new drug approval was obtained in January 2017. Institutional review board approval was obtained in April 2017. Recruitment began in July 2017. Additional funding was approved by the National Center for Advancing Translational Sciences of the National Institutes of Health in August 2018. As of March 2019, enrollment is in progress and expected to be completed by April 2019. Thus far, 10 subjects have been enrolled: 7 subjects have completed the protocol (target of 10) and 3 subjects were withdrawn early by the principal investigator, as they did not meet the eligibility criteria by the time of their first visit. Final data analysis will commence soon after, and the results are expected to be submitted for publication in 2019.

## Discussion

Although intranasal ketamine has been shown to be effective in the treatment of chronic breakthrough pain, there are limited data regarding the use of ketamine as an adjuvant to opioids for the management of *cancer* pain. If intranasal ketamine can be utilized for pain control in patients with cancer, it could provide superior analgesia and a better quality of life without the risk of significant respiratory depression associated with opioid medications. These findings will be an important initial step toward testing the effectiveness of intranasal ketamine as a nonopioid medication for cancer pain and as potential maintenance outpatient therapy.

## Appendix

Multimedia Appendix 1 Numerical pain rating scale.

Multimedia Appendix 2 Side Effects Rating Scale for Dissociative Anesthetics.

Multimedia Appendix 3 Eastern Cooperative Oncology Group performance status.

Multimedia Appendix 4 Edmonton Symptom Assessment System.

Multimedia Appendix 5 The Richmond Agitation-Sedation Scale, used to assess sedation.

Multimedia Appendix 6 The Confusion Assessment Method for the Intensive Care Unit, used to assess for delirium.

Multimedia Appendix 7 Patient Health Questionnaire - 9 items.

Multimedia Appendix 8 Montgomery-Asberg depression scale.

Multimedia Appendix 9 Patient-Reported Outcomes Measurement Information System questionnaire (part 1).

Multimedia Appendix 10 Patient-Reported Outcomes Measurement Information System questionnaire (part 2).

Multimedia Appendix 11 Weekly pain diary. Completed by the subject each week during participation in the study.

Multimedia Appendix 12 Peer-reviewer report #1 from ACTSI/Georgia CTSA KL2 and K12-BIRCWH.

Multimedia Appendix 13 Peer-reviewer report #2 from ACTSI/Georgia CTSA KL2 and K12-BIRCWH.

## Abbreviations

ADL: activities of daily living
AUC_0-t_: total area under-the-concentration-time curve
CAM ICU: Confusion Assessment Method for the Intensive Care Unit
C_max_: peak concentration after each dose and route
DBRCT: double-blind randomized controlled trial
DSMC: Data and Safety Monitoring Committee
ECOG: Eastern Cooperative Oncology Group
ESAS: Edmonton Symptom Assessment
FDA: Food and Drug Administration
MAD: mucosal atomization device
MADRS: Montgomery-Asberg Depression Rating Scale
NMDA: N-methyl-D-aspartate NMDA
NPRS: Numerical Pain Rating Score
PHQ-9: Patient Health Questionnaire - 9 items
PROMIS: Patient-Reported Outcomes Measurement Information System
RASS: Richmond Agitation-Sedation Scale
SERSDA: Side Effect Rating Scale for Dissociative Anesthetics
