# Supplementary figures and images for: Safety of Intranasal Ketamine for Reducing Uncontrolled Cancer-Related Pain: Protocol of a Phase I/II Clinical Trial
Source: JMIR Res Protoc. 2019 Apr 30;8(4):e12125. doi: 10.2196/12125 (PMC6658277; doi:10.2196/12125)

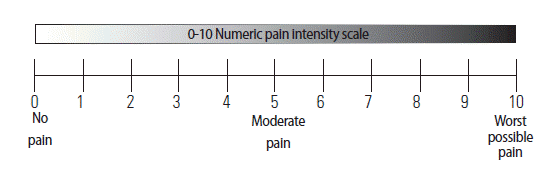

Supplement: Multimedia Appendix 1 [file resprot_v8i4e12125_app1.png]

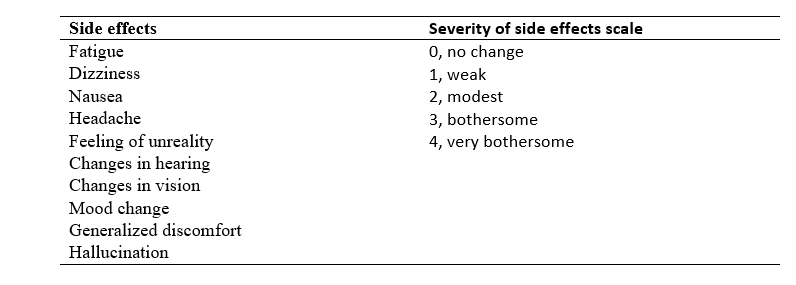

Supplement: Multimedia Appendix 2 [file resprot_v8i4e12125_app2.png]

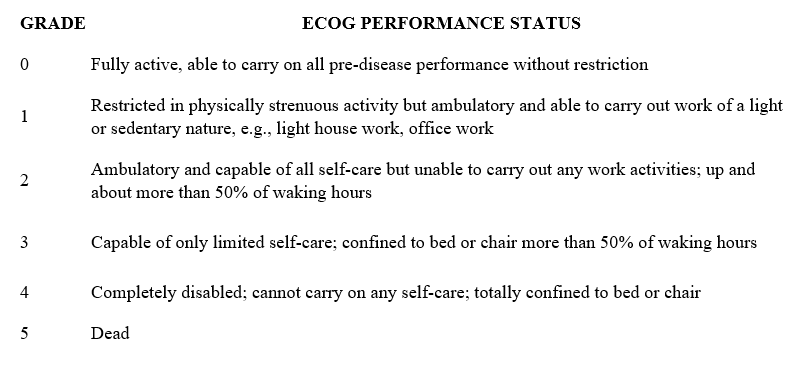

Supplement: Multimedia Appendix 3 [file resprot_v8i4e12125_app3.png]

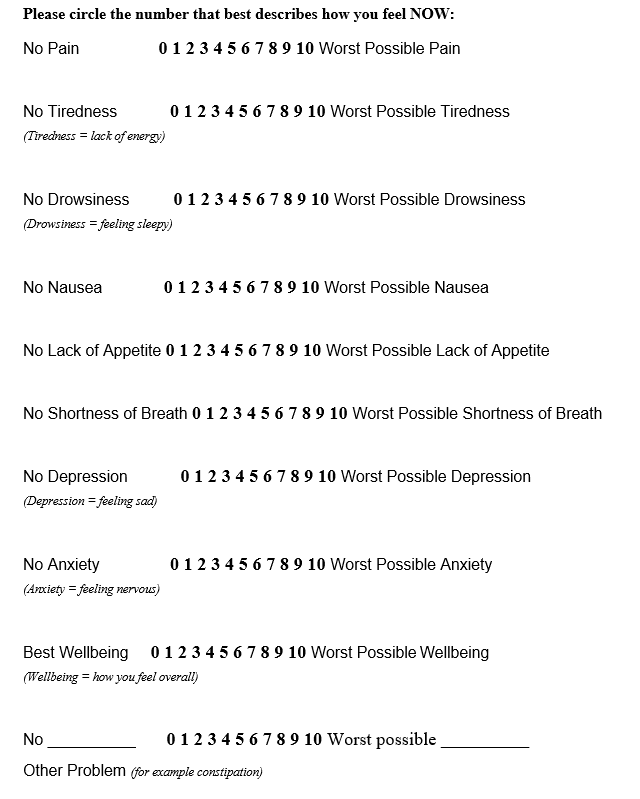

Supplement: Multimedia Appendix 4 [file resprot_v8i4e12125_app4.png]

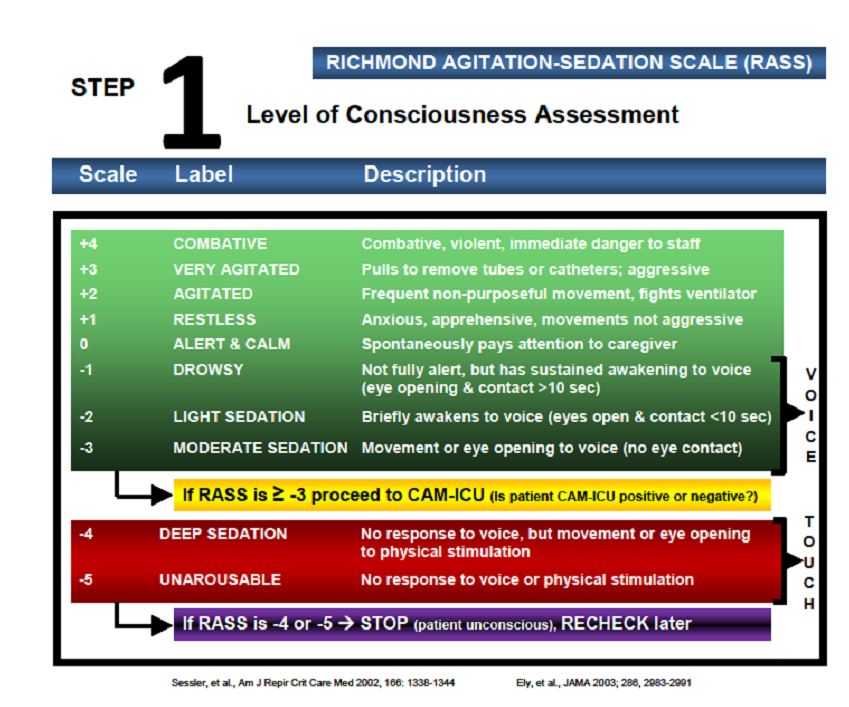

Supplement: Multimedia Appendix 5 [file resprot_v8i4e12125_app5.png]

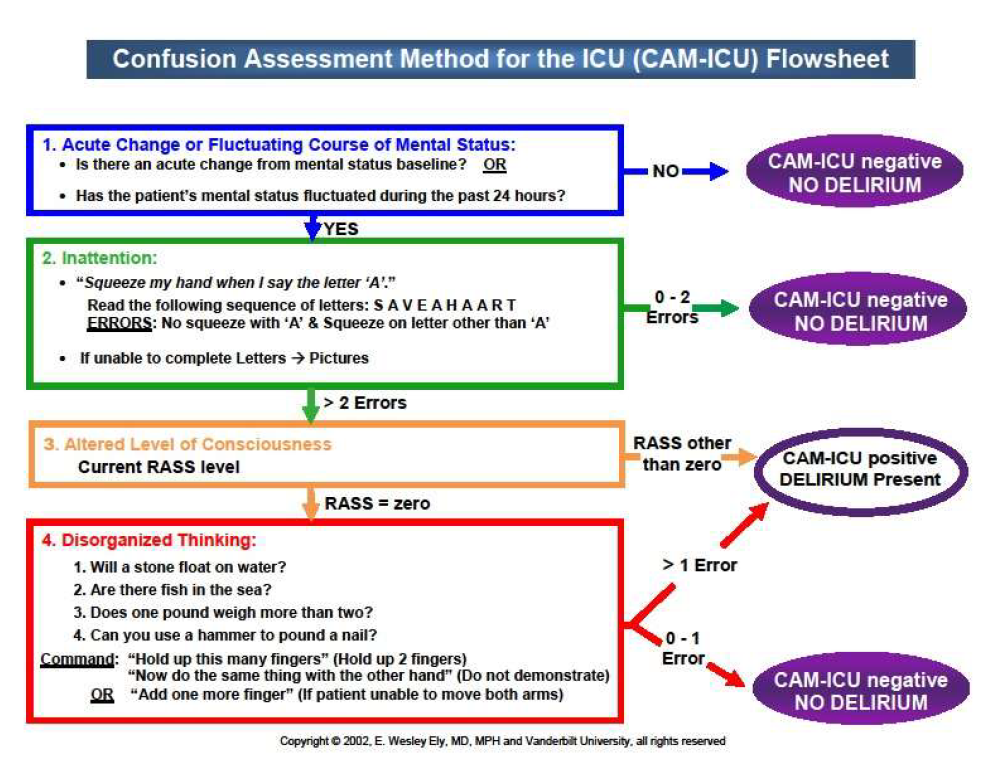

Supplement: Multimedia Appendix 6 [file resprot_v8i4e12125_app6.png]

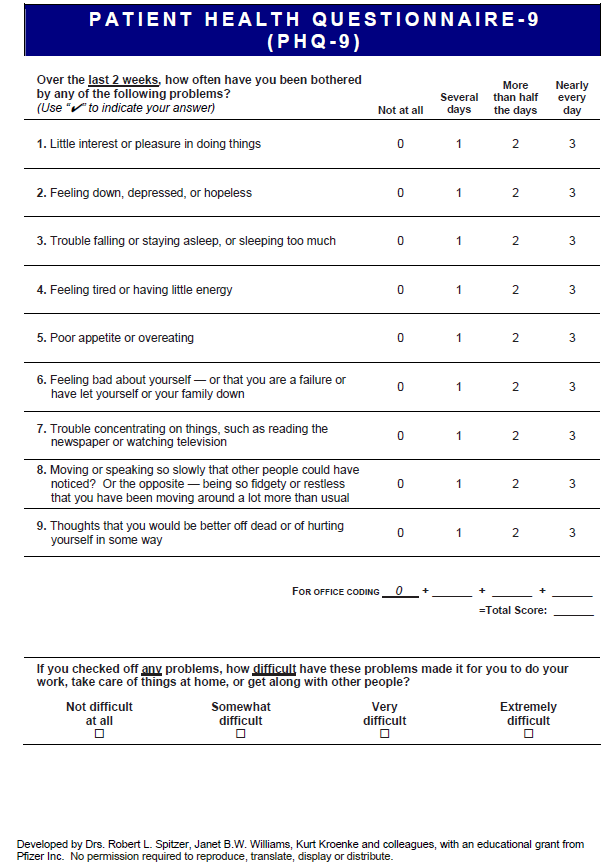

Supplement: Multimedia Appendix 7 [file resprot_v8i4e12125_app7.png]

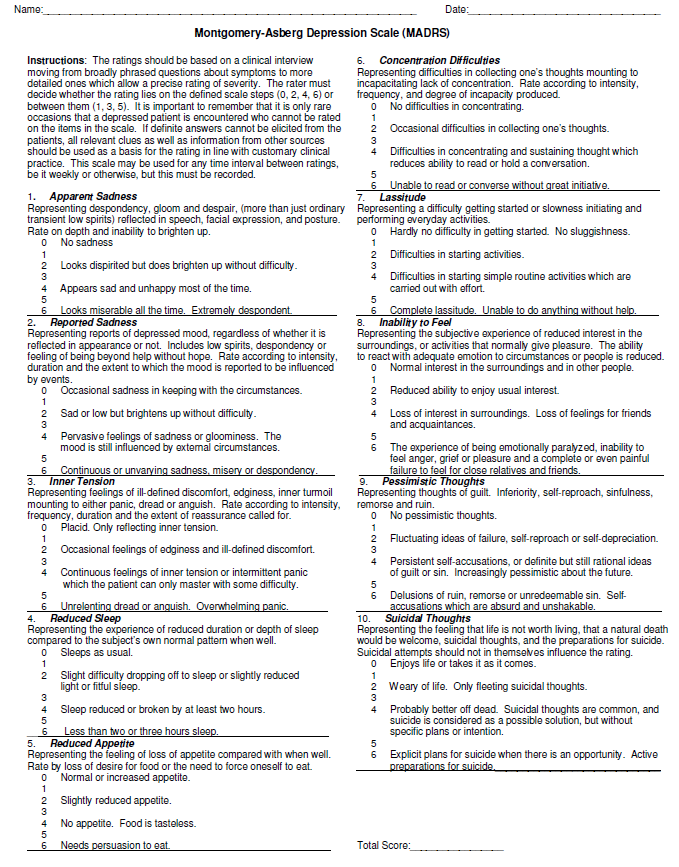

Supplement: Multimedia Appendix 8 [file resprot_v8i4e12125_app8.png]

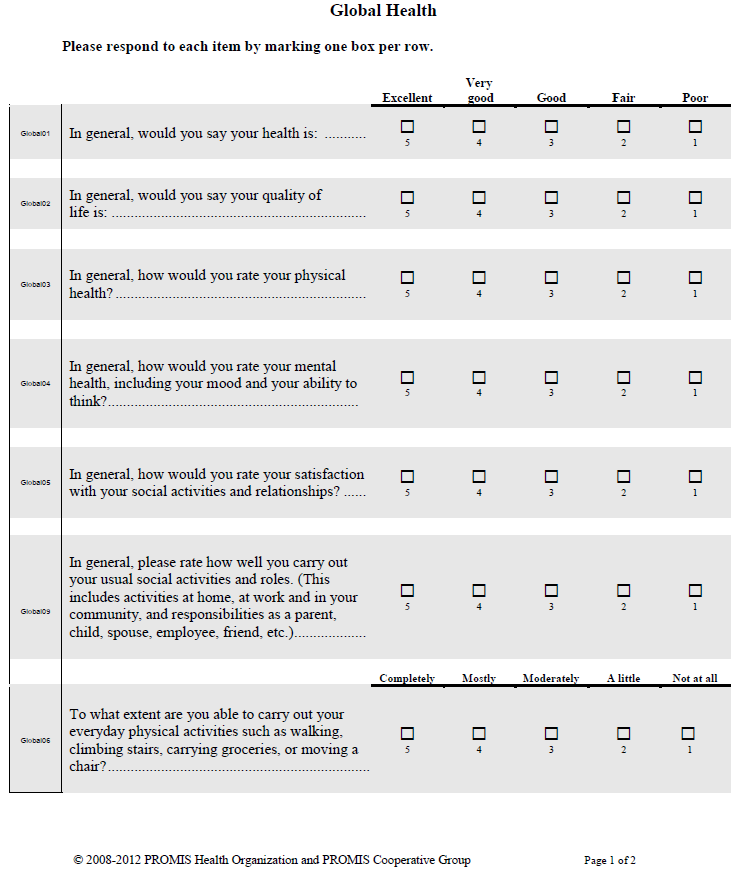

Supplement: Multimedia Appendix 9 [file resprot_v8i4e12125_app9.png]

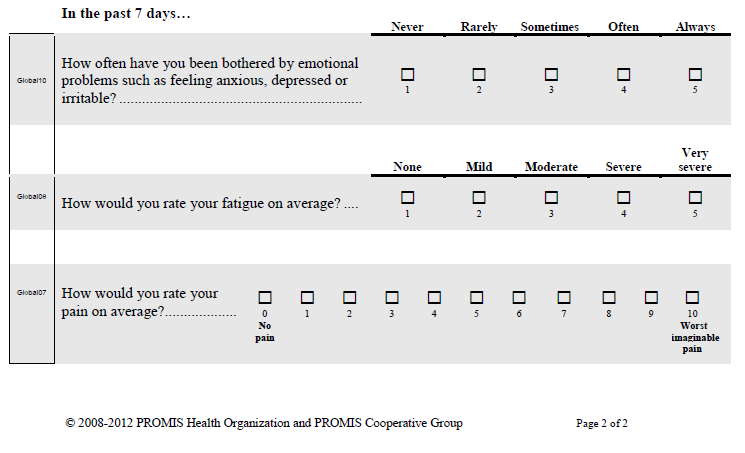

Supplement: Multimedia Appendix 10 [file resprot_v8i4e12125_app10.png]

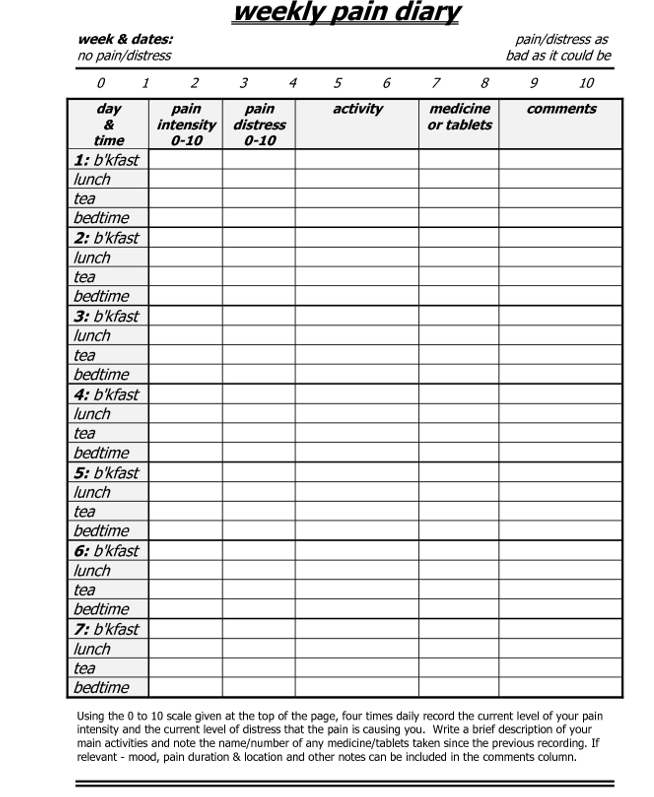

Supplement: Multimedia Appendix 11 [file resprot_v8i4e12125_app11.png]
