# Supplementary material for: Safety of Intranasal Ketamine for Reducing Uncontrolled Cancer-Related Pain: Protocol of a Phase I/II Clinical Trial
Source: JMIR Res Protoc. 2019 Apr 30;8(4):e12125. doi: 10.2196/12125 (PMC6658277; doi:10.2196/12125)
Supplement: Multimedia Appendix 12 [file resprot_v8i4e12125_app12.pdf]

Georgia CTSA KL2 and K12-BIRCWH Review

Applicant: Vinita Singh

Title of Application: Safety, efficacy and feasibility of intranasal ketamine for uncontrolled cancer pain.

Reviewer 1

SCORED REVIEW CRITERIA

Reviewers will consider each of the five review criteria below in the determination of scientific and technical merit, and give a separate score for each. Overall score (1-9)

Reviewers will provide an overall impact score to reflect their assessment of the likelihood for the candidate to maintain a strong research program, in consideration of the following five scored review criteria, and additional review criteria. An application does not need to be strong in all categories to have a major impact.

Overall Impact *Write a paragraph summarizing the factors that informed your Overall Impact score.*
In this research proposal entitled, "Safety, efficacy and feasibility of intranasal ketamine for uncontrolled cancer pain, Dr. Singh aims to conduct a clinical trial among individuals with cancer-related pain to understand the effects of intranasal ketamine on patient reported outcomes and determine the opioid sparing effect of this novel use of ketamine. This is a second resubmission of the original proposal and shows significant improvement and progress from the prior submission.<br><br>As a candidate, Dr. Singh continues to show motivation and persistence in pursuing a research career and continues to make significant strides in moving the project along despite the lack of funding. She has obtained IRB and IND approval for the use on intranasal ketamine and has already started enrollment of subjects in the study. There was a concern in the previous application regarding the lack of publications, the mentorship and advisory team, but she has worked hard to address all of those concerns. With regards to protected time, it appears that she already has obtained approval for additional protected time to pursue MSCR training this fall. With this submission, it is becoming clearer that she does have departmental support, which was a previous concern. Importantly, she has made good progress even without significant resources.<br><br>The research plan itself is very feasible and enrollment has started even without funding at this point. Dr. Singh's training and background optimally positions her to access the many affected patients with cancer-related pain. Her primary mentor has a strong track record of mentorship and her mentorship and advisory team includes expertise in both pharmacology and statistics that will allow for successful completion of the proposed

study. The career development plan is more developed in this resubmission, and her ideas for K23 funding are clearer. Still, specific details on how she plans to turn this study into future funding could be more developed, especially if this turns out that intranasal ketamine is not effective in improving patient reported outcomes or opioid sparing.

SCORED REVIEW CRITERIA

Reviewers will consider each of the five review criteria below in the determination of scientific and technical merit, and give a separate score for each.

1. Candidate

Strengths

- Shows significant commitment to pursuing a research career with this second resubmission
- Has started clinical trial despite lack of funding with IRB approval obtained and enrollment has already started
- Letters of reference speak to her passion and dedication to developing into an independent investigator
- Her training and background in anesthesia and pain leaves her well-poised to investigate alternative therapeutic options for the management of cancer-related pain

Weaknesses

- Appear to be well addressed in this resubmission

2. Career Development Plan/Career Goals & Objectives/Plan to Provide Mentoring

Strengths

- Candidate has continued to pursue career development with her mentors and shows progress with regards to publications
- Has obtained departmental support and resources to pursue MSCR in the fall

Weaknesses

- Career development plan is more developed in this resubmission, but would like to see more details on how a K23 application will be constructed

3. Research Plan

Strengths

- Novel study to the test the efficacy and safety of NAS for the treatment of cancer-related pain and to potentially decrease chronic opioid use
- Plan appears to be very feasible and enrollment has already started
- The candidate appears to have access to a wealth of potentially eligible patients for the study

Weaknesses

- May want to better define exclusion criteria instead of using terms like “severe cardiac disease” which is potentially subjective

4. Mentor(s), Co-Mentor(s), Consultant(s), Collaborator(s)

Strengths

- Strong mentorship team and primary mentor has a long track record of success

Weaknesses

- Her palliative care mentor on the prior submission is no longer included in this proposal for unclear reasons

5. Environment and Institutional Commitment to the Candidate

Strengths

- Has support for protected time

Weaknesses

- Clinical load is significant, but appears to have support and resources for protected time

ADDITIONAL REVIEW CRITERIA

As applicable for the project proposed, reviewers will consider the following additional items in the determination of scientific and technical merit, but will not give separate scores for these items.

- A response for Protections for Human Subjects, Vertebrate Animals, and Biohazards **is required for all applications**
- A response for Inclusion of Women, Minorities, and Children **is required** for Human Subjects Research Applications

Protections for Human Subjects

Comments (if applicable): No issues

Data and Safety Monitoring Plan (Applicable for Clinical Trials Only): No issues

Comments (Required if applicable)

Inclusion of Women, Minorities, and Children (Applicable Only for Human Subjects Research)
Comments (Required if Applicable): No issues

Biohazards (Comments Required if Applicable)

Resubmission

Comments: Second resubmission, and introduction addresses the concerns raised in the prior proposal

Budget
Recommended budget modifications

ADDITIONAL COMMENTS TO APPLICANT

Reviewers may provide guidance to the applicant or recommend against resubmission without fundamental revision.

Additional Comments to Applicant (Optional)
