# Supplementary material for: Safety of Intranasal Ketamine for Reducing Uncontrolled Cancer-Related Pain: Protocol of a Phase I/II Clinical Trial
Source: JMIR Res Protoc. 2019 Apr 30;8(4):e12125. doi: 10.2196/12125 (PMC6658277; doi:10.2196/12125)
Supplement: Multimedia Appendix 13 [file resprot_v8i4e12125_app13.pdf]

ACTSI/Georgia CTSA KL2 and K12-BIRCWH Review

Applicant: Vinita Singh

Title of Application: Safety, efficacy and feasibility of intranasal ketamine for uncontrolled cancer pain

Reviewer #2

SCORED REVIEW CRITERIA

Reviewers will consider each of the five review criteria below in the determination of scientific and technical merit, and give a separate score for each. Overall score (1-9)

Reviewers will provide an overall impact score to reflect their assessment of the likelihood for the candidate to maintain a strong research program, in consideration of the following five scored review criteria, and additional review criteria. An application does not need to be strong in all categories to have a major impact.

Overall Impact *Write a paragraph summarizing the factors that informed your Overall Impact score.*
Applicant's primary career goal is to become an independent NIH-funded clinical researcher investigating non-opioid medication and alternative therapy options for pain. In the current project, the applicant proposes to determine the safety, tolerability, and utility of a novel compound and route, intranasal ketamine. Based on the applicant's preliminary data and the existing literature, it is hypothesized that the use of intranasal (NAS) ketamine (a noncompetitive, antagonist of N-methyl-D-aspartate [NMDA] receptors that blocks the NMDA channel in the open state by binding to the phencyclidine [PCP] site located within the lumen of the channel) will be an exciting approach to reduce uncontrolled cancer related pain as an outpatient, needle-free, patient-friendly maintenance therapy. For subsequent extramural funding, the applicant proposes to prepare an application to submit to the NIH K23 Mentored Patient-Oriented Research Career Development program and independent research funding later on.<br>Specific aims of the project (as provided by the applicant) include: 1) To conduct a clinical investigation of NAS ketamine in a sample of patients with cancer related pain. 2) To evaluate effects of NAS ketamine on Patient Reported Outcomes (PROs), such as pain scores, side effects, depression, quality of life, and functional status. 3) To determine opioid sparing effect of NAS ketamine, documenting use of rescue medications and total opioid consumption in relation to the study.<br>This is a resubmission and previous comments have been mostly addressed satisfactorily. The revised application has a clear plan on mentoring, career development and research/clinic trials laid out. Some mentors are in their last year of federal funding, but there are constant funding from the pharma companies that alleviates this concern to some level. Department letter provides the necessary confirmation on 75% protected time if funded or 50% protected time and MSCR admission as an institutional commitment if not funded. Overall, this is a very strong and well written resubmission application with no evident major weaknesses.

SCORED REVIEW CRITERIA

Reviewers will consider each of the five review criteria below in the determination of scientific and technical merit, and give a separate score for each.

1. Candidate

Strengths

- PI is an Assistant Professor in 'Pain Medicine' at the Department of Anesthesiology, Emory University and proposes to attend the MSCR program. Applicant has a MBBS, a medical degree from India.
- PI was engaged in some research and clinical trials previously and has already registered at the clinicaltrial.gov for the proposed study in May 2017.
- PI's department commits 50% protected time for research and MSCR if not funded and 75% protected time if funded with KL2 award
- Has excellent publications record and has recently published a review article on this topic along with the current mentoring team
- PI is experienced in clinical research/trial in this area

Weaknesses

- None noted

2. Career Development Plan/Career Goals & Objectives/Plan to Provide Mentoring

Strengths

- PI is planning to enroll into the Emory MSCR program with the departmental support (if not funded) and this training will be complete by the end of Spring 2019.
- A clear career development plan has been laid out with details on potential publications and future plans on submitting a K23 application to do a Phase III clinical trial in this area.
- PI will seek additional training in pharmacokinetics and pharmacodynamics prior to K23 application preparations.
- Concerns from the previous submission on unclear career development plan has been addressed.
- Training plan by year, as explained in a table is very clear and is feasible

Weaknesses

- None

3. Research Plan

Strengths

- In aim 1, PI will measure pharmacokinetics of NAS ketamine through analysis of ketamine and its metabolite norketamine to determine pharmacokinetic properties. In aim 2, PI will assess patient reported outcomes as measured by the Numerical

Pain Rating Scale (NPRS), Side Effect Rating Scale for Dissociative Anesthetics (SERSDA), Montgomery Asberg Depression Rating Scale (MADRS), and Edmonton Symptom Assessment (ESAS), Eastern Cooperative Oncology Group (ECOG) and Patient Reported Outcome Measurement Information System (PROMIS) scales. In aim 3, PI will document use of rescue medications prior to and during the study and evaluate total opioid consumption prior to and during the study. Overall plan is well designed.

- Although just 2 case studies so far, the preliminary data is supportive.
- Detailed plan for pharmacokinetics and pharmacodynamics analysis.

Weaknesses

- Phase III clinical trials are generally not allowed on NIH K-series award so other type of support for this would be needed; in addition, an NIH K award does not provide adequate support for a phase III trial.
- Study is not very novel since a phase 2 clinical trial has already been performed on patients with chronic pain. This is a minor concern since the idea is novel for its use in cancer patients.
- Another minor concern is that only 2 patients are recruited as of today although the clinical trial has been active since June 2017.
- Placebo arm is still not included in the revised plan although reasons for this are provided.
- Study is not blinded.
- Although has addressed this concern in the response to critique, the proposal lacks a clear expected outcomes and alternative approaches section.

4. Mentor(s), Co-Mentor(s), Consultant(s), Collaborator(s)

Strengths

- Mentoring team include Drs. Theresa W. Gillespie (Professor, Surgery, and Hematology and Medical Oncology), Donald Harvey (Associate Professor, Hematology and Medical Oncology and Director, Phase I Clinical Trials Unit) and Roman Sniecinski (Associate Professor, Anesthesiology). Advisory committee also include Drs. Zhengjia (Nelson) Chen (Associate Research Professor Biostatistics), and Kimberly Curseen (Associate Professor and Director Outpatient Supportive Care) as collaborator. The mentors have multi-year funding from various federal and non-federal sources.
- Primary mentors have excellent history of previous mentoring junior faculty and co-mentor is a great clinical researcher

Weaknesses

- None

5. Environment and Institutional Commitment to the Candidate

Strengths

- Research environment in support of proposed research at the Emory department of Anesthesiology is excellent. Mentors are experienced in this field and the Department chair and the referees have provided highly supportive letters.

Weaknesses

- None
